# One stone for two birds: Successful treatment of splenic artery aneurysm and gastric varices using endoscopic ultrasound-guided coil and glue embolization

**DOI:** 10.1055/a-2715-4383

**Published:** 2025-11-14

**Authors:** Baobao Wang, Guan-Jun Kou, Jing-Ran Su, Ning Zhong

**Affiliations:** 1 91623 Department of Gastroenterology, Qilu Hospital of Shandong University, Jinan, Shandong, China; 2 91623 Shandong Provincial Clinical Research Center for Digestive Disease, Qilu Hospital of Shandong University, Jinan, Shandong, China

A 70-year-old woman with a long-standing history of hepatic cirrhosis presented with an episode of hematochezia that had occurred 4 months prior to admission. During the current hospitalization, an esophagogastroduodenoscopy revealed both esophageal and GOV1-type gastric varices (GV). In addition, contrast-enhanced computed tomography (CT) demonstrated a 2.7 cm saccular splenic artery aneurysm (SAA) adjacent to the splenic hilum, with a neck of 1.2 cm, and also revealed the presence of gastric fundal varices ( Fig. 1 ).

**Fig. 1:**
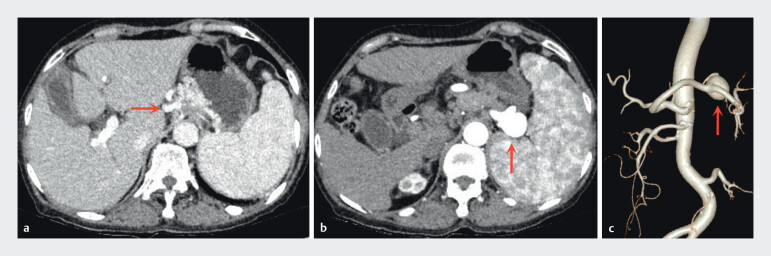
Contrast-enhanced CT images showing **a** gastric fundal varices; **b** a saccular splenic artery aneurysm adjacent to the splenic hilum; **c** a 3D reconstruction of the splenic artery aneurysm.

Endoscopic ultrasound (EUS)-guided cyanoacrylate glue injection was performed for the GV. In the same session, EUS revealed a 2.7 × 1.8 cm SAA originating from the distal splenic artery, with active arterial “to-and-fro” and bidirectional waveform blood flow on Doppler ( Fig. 2 ). Given the proximity of the SAA to the gastric fundus, EUS-guided embolization of SAA was selected. A 19-gauge fine-needle aspiration needle (G31521, Cook Medical, USA) was used to trans-fundus puncture at the SAA and then two 20 × 20 mm Tornado embolization microcoils were deployed, followed by 2 mL of cyanoacrylate glue and 1 mL of distilled water injected. Near-complete obliteration of the SAA was achieved, with patent splenic artery blood flow ( Fig. 3 ). Finally, the endoscopic variceal ligation was conducted for esophageal varices. The total EUS-guided embolization of both GV and SAA was recorded as 28 minutes with no immediate complications ( Video 1 ).

**Fig. 2:**
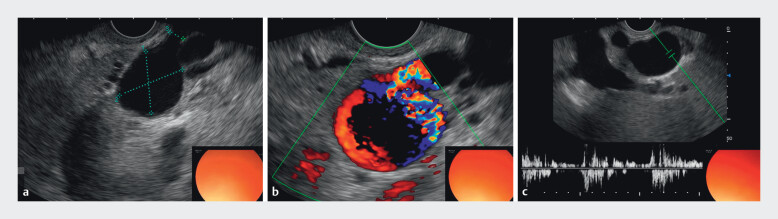
EUS images showing a 2.7 cm × 1.8 cm SAA from the distal splenic artery with bidirectional “to-and-fro” blood flow on Doppler.

**Fig. 3:**
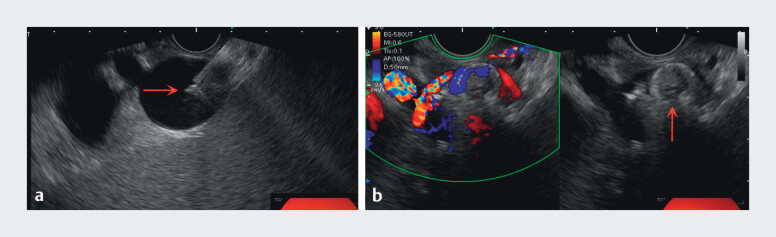
EUS-guided treatment of a splenic artery aneurysm: **a** Injection of cyanoacrylate glue into the aneurysm using a 19-gauge fine-needle aspiration needle and **b** near-complete embolization of the aneurysm.

EUS-guided coil and glue embolization for simultaneous treatment of SAA and gastric varices.Video 1

Postoperative follow-up revealed that the patient was asymptomatic (denying abdominal pain or fever), with stable laboratory parameters (no leukocytosis or thrombocytopenia). Follow-up CT demonstrated optimal coil positioning within the aneurysm and absence of splenic infarction ( Fig. 4 ).

**Fig. 4:**
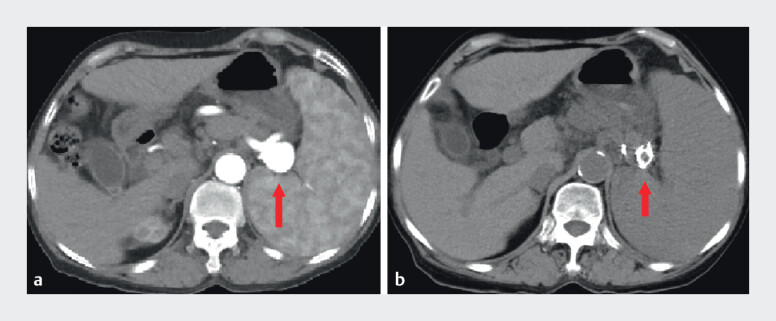
CT images showing **a** SAA prior to treatment and **b** near-complete obliteration of the aneurysm with well-positioned coils after EUS-guided coil and glue embolization.

EUS-guided coil and glue embolization has been previously described and proved effective for visceral arterial pseudoaneurysm 1 2 3 . However, this case demonstrates its successful application in the treatment of SAA, underscoring its potential as a novel, safe, and effective therapeutic alternative for the true aneurysm of the visceral artery.

Endoscopy_UCTN_Code_TTT_1AS_2AB

## Notes

Endoscopy E-Videos https://eref.thieme.de/e-videos
*E-Videos* is an open access online section of the journal *Endoscopy* , reporting on interesting cases and new techniques in gastroenterological endoscopy. All papers include a high-quality video and are published with a Creative Commons CC-BY license. Endoscopy E-Videos qualify for HINARI discounts and waivers and eligibility is automatically checked during the submission process. We grant 100% waivers to articles whose corresponding authors are based in Group A countries and 50% waivers to those who are based in Group B countries as classified by Research4Life (see: https://www.research4life.org/access/eligibility/ ). This section has its own submission website at https://mc.manuscriptcentral.com/e-videos .
